# Identification of cuproptosis-related subtypes, characterization of immune microenvironment infiltration, and development of a prognosis model for osteoarthritis

**DOI:** 10.3389/fimmu.2023.1178794

**Published:** 2023-09-21

**Authors:** Jiao Nong, Guanyu Lu, Yue Huang, Jinfu Liu, Lihua Chen, Haida Pan, Bo Xiong

**Affiliations:** ^1^ Teaching Department, First Affiliated Hospital of the Guangxi University of Chinese Medicine, Nanning, China; ^2^ Postgraduate Schools, Guangxi University of Chinese Medicine, Nanning, China; ^3^ Department of Knee Arthropathy and Sports Injuries, Yulin Orthopedic Hospital of Integrated Traditional Chinese and Western Medicine, Yulin, China

**Keywords:** Osteoarthritis, cuproptosis, prognosis model, tumor immune microenvironment, and subtypes

## Abstract

**Background:**

Osteoarthritis (OA) is a prevalent chronic joint disease with an obscure underlying molecular signature. Cuproptosis plays a crucial role in various biological processes. However, the association between cuproptosis-mediated immune infifiltration and OA progression remains unexplored. Therefore, this study elucidates the pathological process and potential mechanisms underlying cuproptosis in OA by constructing a columnar line graph model and performing consensus clustering analysis.

**Methods:**

Gene expression profifile datasets GSE12021, GSE32317, GSE55235, and GSE55457 of OA were obtained from the comprehensive gene expression database. Cuproptosis signature genes were screened by random forest (RF) and support vector machine (SVM). A nomogram was developed based on cuproptosis signature genes. A consensus clustering was used to distinguish OA patients into different cuproptosis patterns. To quantify the cuproptosis pattern, a principal component analysis was developed to generate the cuproptosis score for each sample. Single-sample gene set enrichment analysis (ssGSEA) was used to provide the abundance of immune cells in each sample and the relationship between these significant cuproptosis signature genes and immune cells.To quantify the cuproptosis pattern, a principal component analysis technique was developed to generate the cuproptosis score for each sample. Cuproptosis-related genes were extracted and subjected to differential expression analysis to construct a disease prediction model and confifirmed by RT-qPCR.

**Results:**

Seven cuproptosis signature genes were screened (DBT, LIPT1, GLS, PDHB, FDX1, DLAT, and PDHA1) to predict the risk of OA disease. A column line graph model was developed based on these seven cuproptosis signature genes, which may assist patients based on decision curve analysis. A consensus clustering method was used to distinguish patients with disorder into two cuproptosis patterns (clusters A and B). To quantify the cuproptosis pattern, a principal component analysis technique was developed to generate the cuproptosis score for each sample. Furthermore, the OA characteristics of patients in cluster A were associated with the inflflammatory factors IL-1b, IL-17, IL-21, and IL-22, suggesting that the cuproptosis signature genes play a vital role in the development of OA.

**Discussion:**

In this study, a risk prediction model based on cuproptosis signature genes was established for the fifirst time, and accurately predicted OA risk. In addition, patients with OA were classifified into two cuproptosis molecule subtypes (clusters A and B); cluster A was highly associated with Th17 immune responses, with higher IL-1b, IL-17, and IL-21 IL-22 expression levels, while cluster B had a higher correlation with cuproptosis. Our analysis will help facilitate future research related cuproptosis-associated OA immunotherapy. However, the specifific mechanisms remain to be elucidated.

## 1 Introduction

Osteoarthritis (OA) is a chronic degenerative joint disease characterized by articular cartilage degeneration, bone fragment formation, subchondral bone remodeling, and synovial inflammation. As the “number one disabling disease” according to the World Health Organization, OA has a significant impact on the quality of life of patients and poses a substantial social and economic burden. Current treatments can only alleviate the symptoms and signs of the disease to a certain extent and delay disease progression; however, treating the etiology of the disease remains challenging.

The synovial membrane is vital in maintaining normal physiological functions of joints and serves as a bridge between the internal structures of the joint and musculoskeletal tissue. The hyaluronic acid secreted by the synovial membrane maintains the integrity of the articular cartilage surface by maintaining its lubrication, thereby reducing friction during joint movement and providing nutrients to other soft tissues within the joint. However, most early studies on OA have focused on chondrocytes, whereas synovial cell lesions were considered secondary to cartilage destruction and have been underappreciated. With the in-depth study of OA, increasing research data have confirmed that OA is a joint disease closely related to synovial tissue and can be induced by the interaction between the synovium and various tissues, such as cartilage and bone, through the secretion of soluble mediators (1). Synovial inflammation is a critical pathological manifestation in the development of OA, as synovial lesions precede cartilage lesions (2, 3). However, the role of synovial lesions in the development of OA remains unclear. Therefore, exploring the pathogenesis and diagnostic markers of OA from the perspective of the synovium is essential to identify novel therapeutic targets for OA, alleviate symptoms, and improve prognosis.

The pathogenesis of OA remains unclear, and relevant studies indicate that OA has a multifaceted etiology (4, 5). Indeed the immune system has been shown to play a crucial role in OA development. More specifically, immune cell infiltration mediates the autoimmune response to OA, thereby inducing the secretion of chemokines, pro-inflammatory cytokines, and proteases, consequently disrupting the immune homeostasis and accelerating OA development (6–8).

Copper ions are essential for maintaining life in microorganisms, plants, non-human animals, and humans, as they function as cofactors for several essential enzymes. Under normal conditions, intracellular copper ion concentrations are maintained at a low level by active homeostatic mechanisms (9). Nonetheless, the gradual accumulation of copper ions above a threshold value leads to excessive respiration of cells and cytotoxicity. copper-dependent controlled cell death in human cells is a novel form of cell death that differs from other known cell death pathways. The authors termed this “cuproptosis,” a process that occurs through the direct binding of copper ions to lipid acylated components of the tricarboxylic acid cycle in mitochondrial respiration, causing lipid acylated protein aggregation and subsequent downregulation of iron-sulfur cluster proteins, thereby promoting proteotoxic stress and ultimately causing cell death (9).

The importance of copper homeostasis in immune infiltration has also been demonstrated in several recent correlative studies (10, 11). Tan et al. (10) observed that copper chelation of macrophages eliminated lysyl oxidase-like 4 presentation-induced internalization of programmed death-ligand 1, thereby inhibiting the immune escape of cells. Meanwhile, Choi et al. (11) reported that chloroiodohydroxyquin (a common copper chelator) is effective in reducing the infiltration of encephalitogenic immune cells (such as CD4 and CD8). This underscores the importance of elucidating the mechanism underlying cuproptosis in immune infiltration. However, no correlations have been reported between cuproptosis-mediated immune infiltration and OA pathology. Therefore, this study sought to elucidate the specific mechanisms underlying cuproptosis-mediated immune infiltration in OA by combining OA microarray data and cuproptosis-related genes.

Recently, bioinformatics and microarray technologies have gained increasing attention and are now widely used in oncology to provide researchers with reliable research directions and theoretical support by predicting disease pathogenesis, diagnosis, and therapeutic targets, making them effective tools for the detection of gene expression, identifying biomarkers, and assessing epigenetic variation. Hence, their scope has rapidly expanded from oncology to OA (12). All OA synovial tissue gene microarray datasets in this study were obtained from the National Center for Biotechnology Information (NCBI) Gene Expression Omnibus database. To investigate the involvement of cuproptosis regulators in the diagnosis and subtype categorization of OA, four sets of gene microarray datasets that satisfied the experimental conditions were obtained.

In this study, a gene prediction model based on seven candidate cuproptosis regulators (*DBT*, *LIPT1*, *GLS*, *PDHB*, *FDX1*, *DLAT*, and *PDHA1*) was developed using the Random Forest (RF) model, Support Vector Machines (SVM) machine learning, and column line graph model. The prediction model was used to predict OA susceptibility, and it was effective in determining the prognosis of patients with OA, thus providing a basis for clinical decision making. Overall, our study can help determine whether cuproptosis molecular subtype patterns can be applied to differentiate between OA cases that are characterized by inflammatory responses and those that are not. Moreover, the findings of this study define the cuproptosis-related genes associated with OA and their correlation with immune cells and inflammation-related factors, thus providing potentially new directions for future OA research.

## 2 Materials and methods

### 2.1 Materials

#### 2.1.1 Data sources

The Gene Expression Omnibus (GEO, http://www.ncbi.nlm.nih.gov/geo/) is a public genomics database for storing gene expression profiles, raw sequences, and platform information. We searched the NCBI GEO database for microarray datasets using the term “osteoarthritis.” Four OA-related datasets, containing 29 and 49 healthy and OA synovial samples, respectively, were downloaded for this study (**Table 1**).

**Table 1:** Characteristics of GEO Datasets Included in the Study.

Number	Series	CON	OA	Platforms
1	GSE12021	9	10	GPL96
2	GSE32317	0	19	GPL570
3	GSE55235	10	10	GPL96
4	GSE55457	10	10	GPL96
		29	49	

### 2.2 Methods

#### 2.2.1 Data integration and pre-processing

In this study, each of the four datasets was annotated using Perl software to map the probes to the platform annotation information, and the names of the probes were converted to gene names to merge the four datasets. The raw data were converted to expression data using the robust multi-array averaging algorithm of the limma package in the R language software (version 4.1.3). The expression levels of the probe sets were translated to gene expression levels by averaging the expression values of several probes for a specific gene using the Bioconductor annotation tool in R. The resulting expression value files containing gene matrices based on the four GEO series were then batch normalized to remove systematic differences among studies, resulting in gene matrix files with row and column names indicating sample and gene names, respectively, for subsequent analysis.

#### 2.2.2 Extraction of cuproptosis-related genes and differential expression analysis

A search on PubMed using the subject term “cuproptosis” revealed 20 cuproptosis-related genes (9). The annotated gene matrix files were secondarily modified to obtain expression matrices of cuproptosis-related genes. Subsequently, the OA-related and significant cuproptosis differential genes (differentially expressed genes) were analyzed and identified using the “reshape2” and “ggpubr” packages in R. The expression data were then converted into ggplot2 input files and used to plot heat maps and box plots.

#### 2.2.3 Constructing and selecting a disease prediction model

RF models, which combine bagging and random feature algorithms, are an effective supervised learning approach that outperform other machine learning algorithms including linear regression models (13). In this study, the RF model was constructed using the “randomforest” package in R software. The eligible genes were selected from 20 cuproptosis-related genes as the independent variables, and patients with OA were used as the response variables to predict the occurrence of OA disease. The SVM is a supervised machine learning technique based on the statistical learning theory notion of structural risk minimization (14). It uses “caret,” “DALEX,” and “kernlab” packages to construct SVM models and plots “residuals reverse cumulative distribution,” “residuals box line plot,” and “subject work characteristic (ROC) curve” for comprehensive evaluation of the model.

#### 2.2.4 Construction of a column line plot model to predict the prevalence of OA

The column line graph prediction model is based on multivariate analysis and integrates multiple predictors according to the personal characteristics of individual patients for achieving an accurate prediction of the probability of something (15). To predict the prevalence of OA based on the prediction model selected in the previous step, we constructed a column line graph model using the “rms” package according to the selected candidate cuproptosis signature genes. To verify the accuracy of the model, “calibration curves,” “decision analysis curves (DCA),” and “clinical impact curves” were plotted to assess the agreement between predicted and actual values. In the present results, the characteristic genes were scored individually in the line graph model, with higher values associated with a higher prevalence of OA, to further assess whether the decisions based on the model are beneficial to the patients.

#### 2.2.5 Genotyping and immuno-infiltration analysis of OA cuproptosis signature genes

A consensus clustering algorithm implemented in the “ConsensusClusterPlus” package provided the number of clusters and their stability (16), which can be used to identify patterns associated with cuproptosis genes. First, only disease group samples were retained, and patients with OA were divided into subgroups with a maximum subgroup classification of k = 9 to select the optimal grouping to investigate the role of cuproptosis signature genes in OA. Subsequently, a PCA was performed to quantify the pattern of cuproptosis and determine whether these groupings were correct. Subsequently, a single sample genomic enrichment analysis (ssGSEA) was performed using the “GSEABase” and “GSVA” packages to quantify the cuproptosis genotyping results and sum the expression levels of these genes by obtaining the ranking of gene expression levels in the samples. By ranking the gene expression levels in the samples, the expression levels were summed to provide the abundance of immune cells in each sample, as well as the relationship between these significant cuproptosis signature genes and immune cells.

### 2.3 Sample collection

Synovial tissue from 3 patients of meniscus injury and 3 of OA were collected from Affiliated Hospital of the Guangxi University of Chinese Medicine. All patients critically read and signed the informed consent form (KY2022-002-02), which was approved by the ethics committee of Affiliated Hospital of the Guangxi University of Chinese Medicine. The research followed the guidelines of the 1975 Declaration of Helsinki.

### 2.4 Reverse-transcription quantitative polymerase chain reaction

The total synovial tissue RNA was extracted using Trizol (Servicebio), and then total RNA was reverse-transcribed to complementary DNA (cDNA) using ServicebioRT Enzyme Mix. The qRT-PCR was performed using the 2×SYBR Green qPCR Master Mix (None ROX) (Servicebio). The primer sequence of genes used in our study is listed in **Table 2**. Genes were normalized to GAPDH. Relative levels of mRNA were expressed as fold-changes as calculated by the 2^®^−ΔΔCT method. Each biological sample was technically performed in triplicate.

**Table 2:** Primer sequences of DBT, LIPT1, GLS, PDHB, FDX1, DLAT and PDHA1.

Gene name	Forward primer	Reverse primer
DBT	CAGTTCGCCGTCTGGCAAT	CCTGTGAATACCGGAGGTTTTG
LIPT1	CCTCTGTTGTAATTGGTAGGCAT	CTGGGGTTGGACAGCATTCAG
GLS	AGGGTCTGTTACCTAGCTTGG	ACGTTCGCAATCCTGTAGATTT
PDHB	AAGAGGCGCTTTCACTGGAC	ACTAACCTTGTATGCCCCATCA
FDX1	TTCAACCTGTCACCTCATCTTTG	TGCCAGATCGAGCATGTCATT
DLAT	CGGAACTCCACGAGTGACC	CCCCGCCATACCCTGTAGT
PDHA1	TGGTAGCATCCCGTAATTTTGC	ATTCGGCGTACAGTCTGCATC

### 2.5 Statistical analysis

All statistical analyses in our study were performed with R software, version 4.2.1. For all figures: * represents *p* < 0.05, ** represents *p* < 0.01, and *** represents *p* < 0.001.

## 3 Results

### 3.1 Differential analysis of cuproptosis genes

**Figure 1:**
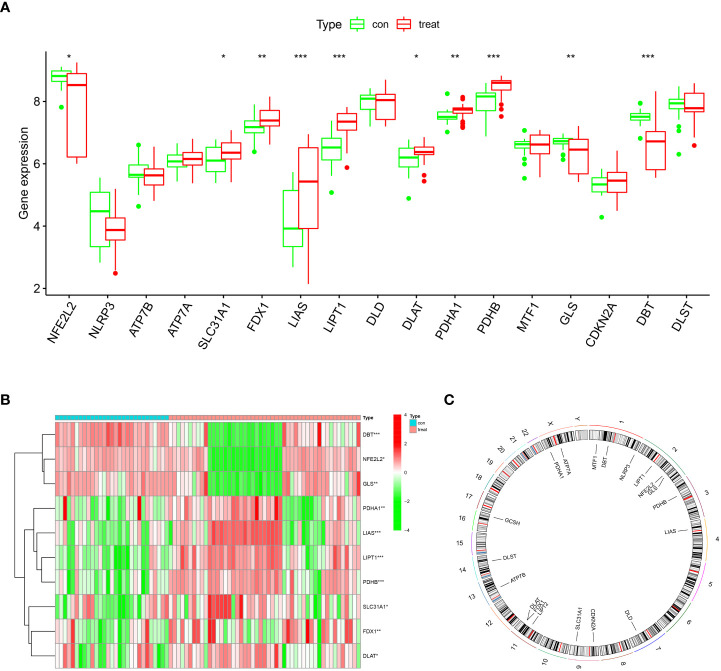
Landscape of cuproptosis regulators in OA **(A)** Box plot of differential expression of genes characteristic of cuproptosis in the OA disease and normal groups. The horizontal coordinates represent the name of the gene, and the vertical coordinates indicate the expression of the gene; blue and red represent the control and disease groups, respectively. **(B)** Heat map of differential expression of ten cuproptosis differential genes in the OA disease and normal groups. **(C)** Positions of ten cuproptosis differential genes in chromosomes. *p < 0.05, **p < 0.01, ***p < 0.001.

Ten cuproptosis signature genes closely related to OA were screened using *t*-test analysis to analyze the differences in expression of cuproptosis-related genes between OA and non-OA patients. *GLS* and *DBT* were lowly expressed in patients with OA (**Figure 1**).

### 3.2 Model construction and selection

**Figure 2:**
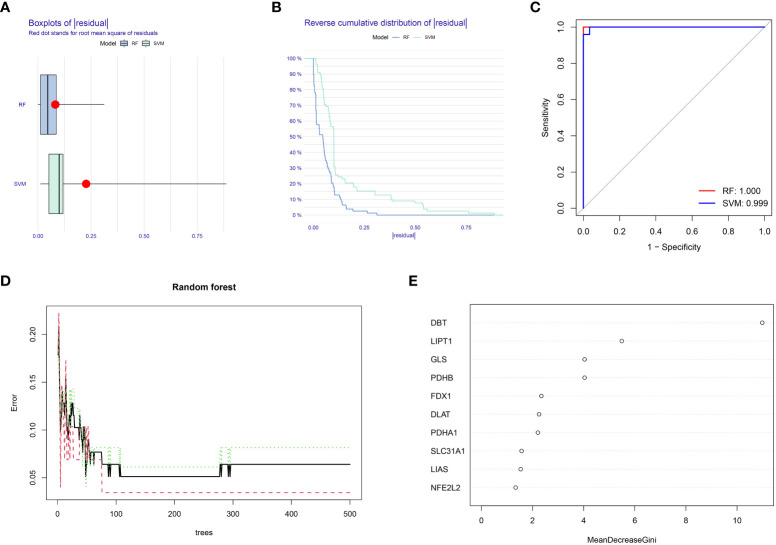
Random forest (RF) model construction. **(A)** Residual box plot depicts the distribution of residuals for the RF and SVM models; lower values of residuals indicate that the model is more meaningful and accurate. **(B)** The inverse cumulative distribution of residuals displays the distribution of residuals for the RF and SVM models. **(C)** ROC curves for the RF and SVM models: higher AUC values indicate higher accuracy of the model. **(D)** Random forest tree plot: horizontal coordinate represents the number of random forest trees, and the vertical coordinate represents the error of cross-validation; the error of the sample of patients with OA is highlighted in red, the error of all samples is shown in black, and the sample of non-OA patients is shown in green. **(E)** Characteristic gene importance score graph: the horizontal coordinate is the score value, and the vertical coordinate is the gene name; the score is directly related to the importance of the characteristic gene.

In this study, RF and SVM models were constructed from ten cuproptosis differential genes for predicting the occurrence of OA disease. Both the “residual box line plot” (**Figure 2A**) and the “residual reverse cumulative distribution plot” (**Figure 2B**) indicated that the RF model had the smallest residuals, thereby substantiating the selection of the RF model for predicting the occurrence of OA disease. The receiver operating characteristic (ROC) curves’ area under the curve (AUC) values (**Figure 2C**) also shows that the RF model was more accurate than the SVM model. In the next step of this study, the RF model was used as a baseline to screen for disease signature genes, and the minimum value of the cross-validation error in the RF tree plot (**Figure 2D**) was used to determine the representative value of trees in the optimal RF tree. Furthermore, the importance scores of the signature genes were plotted (**Figure 2E**). The importance of the genes was determined by the level of the score, and signature genes with a score >2 were selected for subsequent analysis.

### 3.3 Construction of a column line graph model to predict the prevalence of OA

**Figure 3:**
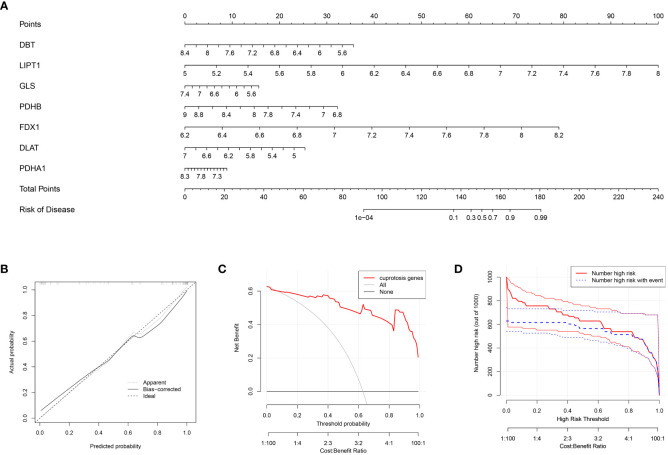
Construction of the nomogram model **(A)** Column line graph model constructed based on the cuproptosis signature gene: calibration curve at the top of the column line graph demonstrates the accuracy of the model. **(B)** Calibration curve of the column line graph: x-axis indicates the probability predicted by the column line graph, while the y-axis indicates the likelihood of actual OA disease. A closer distance between the solid and dashed lines indicates higher accuracy of the model. **(C)** DCA curve indicates that the columnar line graph model has a higher clinical value than individual characteristic genes and is more meaningful for the assessment of disease prediction in patients with OA. **(D)** Clinical impact curves: used to assess the clinical impact of the line plot model; red indicates patients classified as high risk, while blue indicates true positive patients.

In this study, the cuproptosis signature genes obtained from the previous screening step regarding OA with gene scores >2 were used to construct a column line graph model (**Figure 3A**) to obtain a score for each gene in OA disease. The scores for these signature genes were summed to predict the prevalence of OA based on them. The calibration curve (**Figure 3B**) suggests that this column line graph model diagnoses OA positivity in line with the actual positivity rate. DCA graph (**Figure 3C**) revealed that the red line consistently stayed above the gray-black line between 0 and 1, which suggests the significance of the column line graph model for predicting the occurrence of OA disease based on its high clinical application. Similarly, the clinical impact curve (**Figure 3D**) predicts that the column line graph model has strong predictive power.

### 3.4 Genotyping analysis

**Figure 4:**
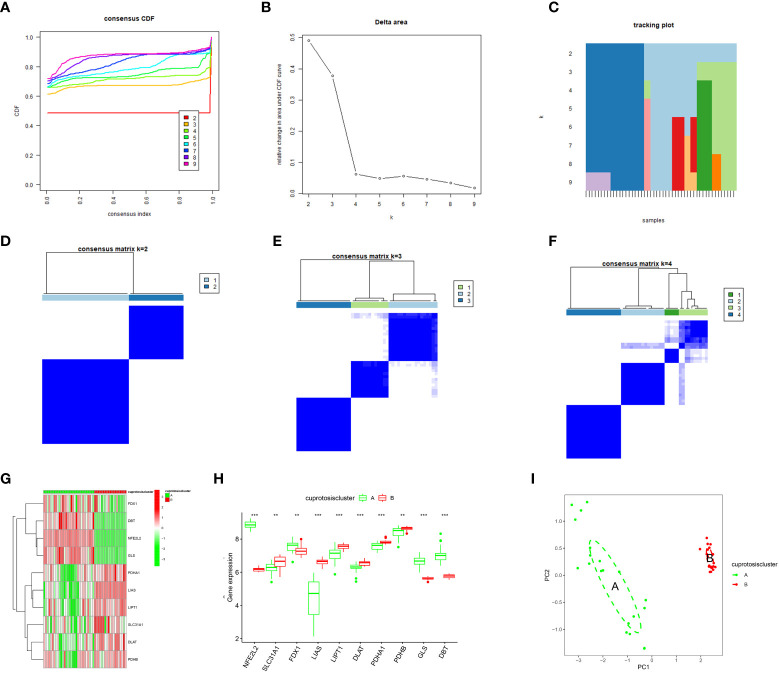
Consensus clustering of the ten significant cuproptosis regulators in OA. **(A)** Cumulative distribution function (CDF) of consistent clustering for k = 2–9. **(B)** Area fraction under the CDF curve for k = 2–9. C Trace plot for k = 2–9. **(D–F)** Heat map of scale matrix in the OA sample. **(G)** Heat map of consensus clustering of the ten cuproptosis trait genes in the two populations. **(H)** The box-line plot of consensus clustering of the ten cuproptosis signature genes in the two populations. **(I)** PCA analysis: blue for CA, red for CB, demonstrating significant differences in genes between patterns. *p < 0.05, **p < 0.01, ***p < 0.001.

This study performed a consensus cluster analysis of OA samples based on the expression of ten cuproptosis signature genes to investigate the pattern of modifications in OA cuproptosis (**Figures 4A, B**). Results revealed that, among them, the cumulative distribution function (CDF) values were the smallest when k = 4. However, the correlation between groups was high when patients with OA were divided into 3 or 4 groups. Therefore, two different OA subtypes (clusters A and B) could be identified by analyzing the differential expression of these cuproptosis signature genes (**Figures 4C–F**). Heat maps and box line plots were plotted to illustrate the differential expression levels of these cuproptosis signature genes between the two populations. The heat map and box line plots in the communities (**Figures 4G, H**) depict that *SLC31A1*, *LIAS*, *LIPT1*, *DLAT*, *PDHA1*, and *PDHB* are more highly expressed in CB than in CA, whereas *NFE2L2*, *FDX1*, *GLS*, and *DBT* are less highly expressed in CB than in CA. PCA analysis of the two isoforms (**Figure 4I**) revealed that the ten cuproptosis signature genes could be distinguished between two completely different patterns and significant differences existed between the clusters.

### 3.5 Immune infiltration analysis

**Figure 5:**
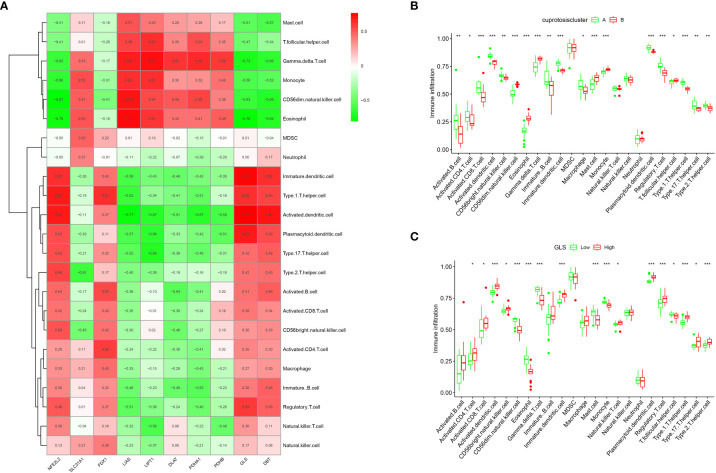
Single sample gene set enrichment analysis. **(A)** ssGSEA analysis results: horizontal coordinates represent associated immune cells; vertical coordinates represent immune infiltration. **(B)** Immune cell correlation analysis: each cell corresponds to the correlation coefficient between the gene and immune cells; positive and negative correlations are marked in red and blue, respectively. **(C)** Box plot of the analysis of differences in immune cells in the high- and low-GLS expression groups (*p < 0.05, **p < 0.01, ***p < 0.001).

The ssGSEA enrichment analysis revealed that patients in the CA group highly expressed activated B cells, activated CD4 T cells, activated CD8 T cells, activated dendritic cells, and CD56 high-expressing natural killer cells (**Figure 5A**). In contrast, patients in the CB group were highly expressed mainly in CD56 low-expressing natural killer cells, eosinophils, and γδ T cells. We also assessed its correlation with immune cells (**Figure 5B**) and observed that GLS was positively correlated with many immune cells. Subsequently, the difference in immune cell infiltration correlation between high and low GLS expression in patients with OA was further explored (**Figure 5C**), and the results indicated that patients with OA with high GLS expression exhibited enhanced immunocompetence.

### 3.6 Identification of cuproptosis gene patterns and signature genes

**Figure 6:**
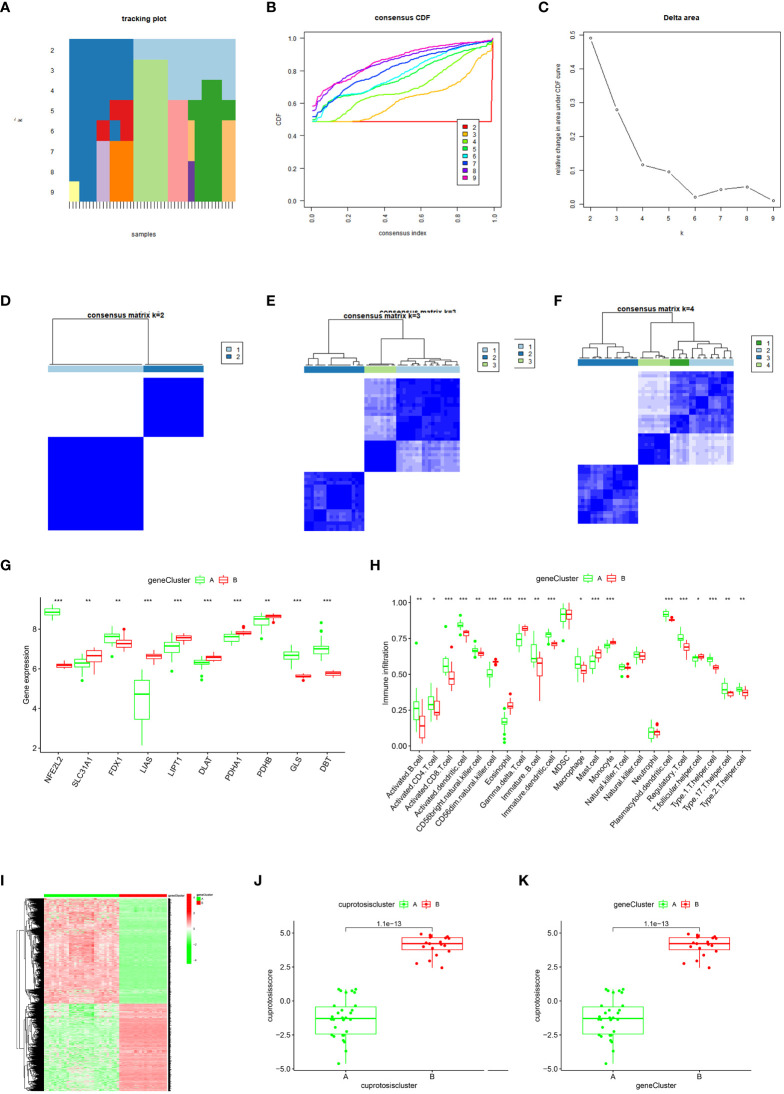
Consensus clustering of the cuproptosis regulators-related DEGs in OA.]**(A–F)** Consistency matrix plot of the ten cuproptosis-associated DEGs for k = 2–5. **(G)** Box-line plot of differential expression of ten key cuproptosis genes in gene clusters A and **(B, H)** Differential immune cell infiltration between gene clusters A and **(B, I)** Heat map of the expressiveness of the ten cuproptosis-associated DEGs in gene clusters A and B **(J–K)** Differences in cuproptosis scores between Cluster A and Cluster B (*p < 0.05, **p < 0.01, ***p < 0.001).

To further validate the gene pattern of cuproptosis, we used a consensus clustering approach to classify patients with OA into different genomic subtypes based on 20 cuproptosis-associated DEGs. We determined that two distinct cuproptosis gene patterns (gene cluster A and gene cluster B) existed, consistent with the grouping of cuproptosis patterns (**Figures 6A–F**). **Figures 6G, H** confirms that the differential expression levels of the ten cuproptosis signature genes, as well as the immune cell infiltration between gene clusters A and B, are similar to the cuproptosis pattern results, thereby validating the accuracy of the consensus clustering approach for grouping. The expression levels of the 20 cuproptosis-associated DEGs in gene clusters A and B are shown in **Figure 6I**. To quantify the cuproptosis pattern, we calculated the corresponding cuproptosis scores for each sample between the two different cuproptosis patterns or cuproptosis gene patterns using the PCA algorithm, which suggested (**Figures 6J, K**) that cluster B or gene cluster B had a higher cuproptosis score than cluster A and gene cluster A.

### 3.7 Role of cuproptosis patterns in OA

**Figure 7:**
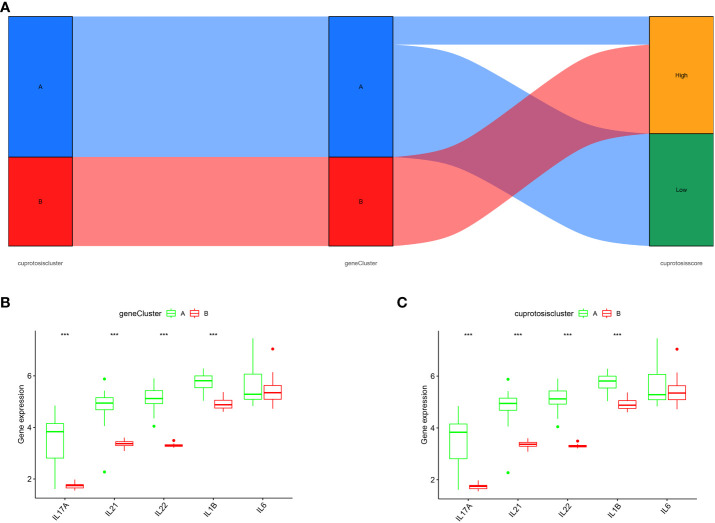
Role of cuproptosis patterns in distinguishing OA. **(A)** Sankey diagram depicting the relationship between cuproptosis typing results, cuproptosis genotyping results, and high- and low-cuproptosis gene scores. **(B)** Differential expression levels of IL-17A, IL-21, IL-22, IL-1β, and IL-6 between cuproptosis typing Cluster A and Cluster B. **(C)** Differential expression levels of IL-17A, IL-21, IL-22, IL-1β, and IL-6 between gene clusters A and B; expression levels of IL-17A, IL-21, IL-22, IL-1β, and IL-6 are shown (***p < 0.001).

The relationship between cuproptosis patterns, cuproptosis gene patterns, and cuproptosis scores is presented as a Sankey diagram (**Figure 7A**). We investigated the relationship between cuproptosis patterns and OA *via* exploring the correlation between cuproptosis and interleukin (IL)-17A, IL-21, IL-22, IL-1, and IL-6. The results revealed that IL-17A, IL-21, IL-22, and IL-1 expression levels were higher in cluster A or gene cluster A than in cluster B or gene cluster B, suggesting that cluster A or gene cluster A is highly correlated with OA characterized by inflammatory responses (**Figures 7B, C**).

### 3.8 Validation of hub genes

**Figure 8:**
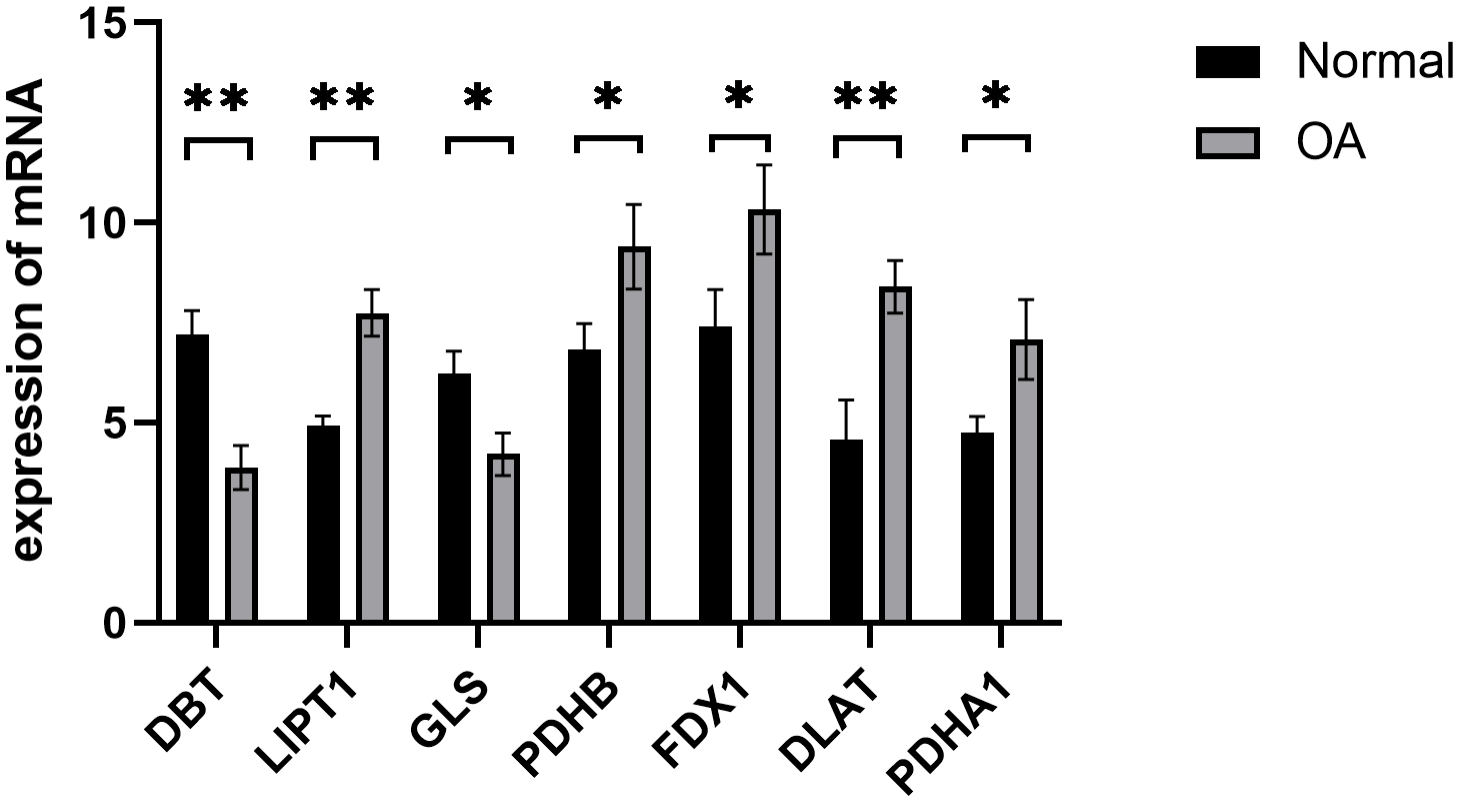
Validation of hub cuproptosis genes using qRT-PCR. The relative mRNA expressions of *DBT*, *LIPT1*, *GLS*, *PDHB*, *FDX1*, *DLAT*, and *PDHA1* were displayed (*p < 0.05, **p < 0.01).

We confirmed the seven cuproptosis-related biomarkers using RT-qPCR in order to verify our results. In comparison with the control group, the expression of DBT and DLS were down-regulated in OA synovial tissue; however, the expression of LIPT1, PDHB, FDX1, DLAT, and PDHA1 were significantly up-regulated (**Figure 8**). These results were consistent with our predictions using bioinformatics tools.

## 4 Discussion

Cuproptosis is a recently discovered form of cell death characterized by the accumulation of free copper in cells and lipidation of proteins leading to cytotoxic stress, which induces cell death (9). However, data on the mechanism underlying cuproptosis in OA remain limited. Therefore, we performed the first study to explore the prognostic role of cuproptosis-related genes in patients with OA using a comprehensive gene expression database. Differential expression analysis was performed between OA and non-OA patient samples, and RF and SVM models were developed to predict the prevalence of OA. Based on the superiority of the RF model, we constructed a predictive column using which a short-term treatment plan could be developed for patients. The DCA curve and clinical impact curve analyses indicated that the model has unique clinical diagnostic advantages. We ultimately identified seven cuproptosis signature genes associated with OA: DBT, LIPT1, GLS, PDHB, FDX1, DLAT, and PDHA1. RT-qPCR was performed to verify our findings, and it was consistent with results from bioinformatics tools, which reaffirmed the important role of cuproptosis-related biomarkers in OA.

DBT regulates the degradation of branched-chain amino acids and is the transacylase component of the mitochondrial multienzyme branched-chain α-keto acid dehydrogenase complex (17). DBT induces proteotoxic stress, which ultimately leads to cellular cuproptosis by regulating the process of protein–lipid acylation metabolism, thereby leading to lipid acylated protein aggregation and subsequent downregulation of iron-sulfur cluster protein expression (9).

*LIPT1* is a vital gene encoding the mitochondrial lipoic acid pathway (9). Abdul et al. (18) reported that lipoic acid reduces obesity-induced inflammation in the body by modulating the immune system when chronic treatment with lipoic acid agonists is administered. In addition, the synthesis of mitochondrial fatty acids is inextricably linked to LIPT1 expression (19). Osteoblast samples from OA secreted more pro-inflammatory cytokines when induced by free fatty acids, whereas the expression of signaling molecules from related pathways was not altered. This suggests that the participation of free fatty acids in subchondral bone damage may be more dependent on the inflammatory response and immune system than on related signaling pathways (20).

PDHB and PDHA1 are essential for maintaining normal mitochondrial metabolism and are directly or indirectly involved in the mitochondrial tricarboxylic acid cycle. Moreover, their binding to copper ions leads to the accumulation of lipid-acylated proteins, resulting in mitochondrial metabolic dysfunction and, consequently, inducing cell death (21, 22). PDHB and PDHA1, two E1 isoforms of the pyruvate dehydrogenase complex, are located mainly in the mitochondria of cells and catalyze the conversion of glucose-derived pyruvate to acetyl coenzyme A (23–25), which promotes the expression of inflammatory cytokines through the regulation of histone acetylation (26). Moreover, an OA-related animal study also showed that excessive accumulation of acetyl coenzyme A, stimulated by matrix metalloproteinases, further exacerbated inflammation in OA mice (27). In addition, the pyruvate dehydrogenase complex is an essential part of the tricarboxylic acid cycle (28).

The acetyl coenzyme A derived from it combines with oxaloacetate to produce citric acid, which is added to the tricarboxylic acid cycle (29, 30). A recent study showed that glucocorticoids can regulate macrophage inflammatory activation and anti-inflammatory polarization by promoting the tricarboxylic acid cycle (31). Additionally, citric acid can be converted to itaconic acid (3, 32), which is an anti-inflammatory metabolite that inhibits succinate oxidation mediated by succinate dehydrogenase (32, 33) and activates anti-inflammatory transcription factors (34). This suggests that drug-targeted modulation of the tricarboxylic acid cycle and acetyl coenzyme A production, particularly the biological activity of PDHB and PDHA1, may be a new anti-OA strategy.

Similar to PDHB, which regulates cuproptosis, GLS is involved in the mitochondrial tricarboxylic acid cycle by modulating the formation of the pyruvate dehydrogenase complex. Upon combination with copper ions, this leads to the aggregation of lipidated proteins, thereby resulting in mitochondrial metabolic dysfunction and inducing cellular cuproptosis (9).

*FDX1* encodes a small iron-sulfur protein involved in reducing mitochondrial cytochromes and the synthesis of various steroid hormones (35). Tsvetkov et al. (9) reported FDX1 as a crucial regulator of cuproptosis by reducing Cu^2+^ to the more toxic Cu^+^, thereby leading to cytotoxic stress and inducing cellular cuproptosis. It is also an upstream regulator of protein–lipid acylation and knockdown of FDX1, a critical upstream regulator of lipid acylated proteins or lipid acylation-related enzymes and can block cuproptosis (9). In a recent lung cancer study, knockdown of FDX1 improved mitochondrial metabolic dysfunction, thereby reducing the inflammatory response of the body (36).

The binding of DLAT mitochondrial proteins to lipidated proteins leads to oligomerization of DLAT lipid acylation, thereby aggregating lipidated proteins and subsequent downregulation of iron-sulfur cluster protein expression, which induces proteotoxic stress and ultimately leads to cellular cuproptosis (9). Colliou et al. (37) reported that *DLAT* expression significantly increased intestinal Th17 cells, thereby preventing inflammatory diseases. Collectively, a close association between OA and *LIPT1*, and *PDHB* and *PDHA1* is implied. However, no relationship between cuproptosis regulators and OA has been reported, and the results of this study will help to better explain of the role of cuproptosis in OA and may provide new directions for future research.

In the early stages of OA pathogenesis, the falling of cartilage fragments into the joint activates the immune cells, which are then recruited to the synovial tissue to produce inflammatory mediators, such as IL-1β, IL-17, and IL-21. Therefore, inflammatory factors infiltrate synovial tissue in patients with OA, and the severity of synovitis is closely related to OA symptoms and disease progression (38). Th17 cells can affect chondrocytes and synovial cells as well as increase production of various enzymes. The concentration of Th17 cells is increased in synovial fluid, synovium, cartilage, and serum during the inflammatory phase of OA, during which they selectively secrete pro-inflammatory factors, such as IL-17A, IL-21, and IL-22, which collectively mobilize, recruit, and activate neutrophils and participate in the inflammatory response (39, 40). The main effector of Th17 cells is IL-17, which is referred to as IL-17A, the first member of the IL-17 family (41). IL-17, an inflammatory cytokine primarily produced by activated T cells (42), can encourage T cell activation and the production of several cytokines, including IL-6 and IL-8, which promote granulocyte-macrophages differentiation, activation, or recruitment. When bound to receptors, pro-inflammatory cytokines mediate the inflammatory response in OA through the MAP kinase pathway and nuclear transcription factor-kappa B pathway. Hence, IL-17A is an early initiator of the T cell-induced inflammatory response and can exacerbate the inflammatory response (43–45). Askari et al. (46) reported that IL-17A levels were considerably higher in patients with OA than in healthy controls, where elevated IL-17A levels may be associated with reduced vitamin D3 levels, and IL-17A levels were positively correlated with Western Ontario and McMaster Universities Arthritis Index (WOMAC) pain scores. IL-21 levels were higher in patients with OA than in healthy controls. Notably, IL-21 expression was positively correlated with disease progression in OA. In the synovial membrane of osteoarthritic patients, IL-21 activates T cells, B cells, and NK cells, thereby promoting the release of inflammatory factors and exacerbating OA (42, 47). IL-22 is mainly secreted by Th22 cells, Th17 cells, and NK22 cells and further promotes inflammation by promoting IL-6 and IL-8 secretion by synovial fibroblasts, endothelial cells, and other cells (48), and stimulates IL-1 and TNF-α secretion by macrophages and mast cells in the periphery and joints. It induces cells to produce vascular endothelial growth factors to promote intra-articular vascular proliferation and induce synovial and joint inflammation (49). Intra-articular vascular proliferation and induced synovial cells produce matrix metalloproteinases, thereby degrading the intra-articular extracellular matrix and participating in the pathogenesis of OA (49–53). IL-1β reportedly promotes the differentiation of Th17 cells (40), and although IL-1β is considered a critical pro-inflammatory cytokine, its absence accelerates the progression of OA in a mouse model (54). Reactive oxygen species, which produce free radicals that aid in cartilage deterioration, are also produced by IL-1 (55). In addition, IL-1β can modulate acute-phase related proteins during the acute phase of OA development, thereby acting as an anti-inflammatory and immunosuppressive agent (56, 57).

The specific molecular mechanism of IL-1β in OA remains controversial. Therefore, further in-depth studies on the specific mechanism of action of IL-1β in OA are warranted. In the present study, two cuproptosis patterns (clusters A and B) were identified using a consensus clustering approach based on ten crucial cuproptosis regulators. Both classification patterns could be effectively distinguished into two clusters, suggesting two completely different subtypes in patients with OA. Cluster A is highly correlated with the Th17 immune response and has higher levels of IL-1, IL-17, IL-21, and IL-22 expression.

This study has certain limitations. First, although the sample size of the microarray data used is sufficient for the study, the sample size may still be small enough to cause some bias in the results, Second, although the cuproptosis genes related to OA have been screened, the specific mechanism of action remains to be elucidated.

Although the current research results cannot fully illustrate the causal relationship between cuproptosis-related genes in the immune regulation of OA, it can be speculated based on previous findings and the results of this study that cuproptosis genes play a crucial role in the immune infiltration of OA. However, the correlation between cuproptosis and immune and inflammatory factors remains to be assessed. Nonetheless, the present study establishes the relationship between cuproptosis patterns and OA and inflammatory factors and predicts some potential directions in the future clinical management of OA.

## 5 Conclusions

In this study, a risk prediction model based on cuproptosis signature genes was established for the first time, and accurately predicted OA risk. In addition, patients with OA were classified into two cuproptosis molecule subtypes (clusters A and B); cluster A was highly associated with Th17 immune responses, with higher IL-1β, IL-17, and IL-21 IL-22 expression levels, while cluster B had a higher correlation with cuproptosis. Our analysis will help facilitate future research related cuproptosis-associated OA immunotherapy. However, the specific mechanisms remain to be elucidated.

## Data availability statement

The datasets presented in this study can be found in online repositories. The names of the repository/repositories and accession number(s) can be found in the article/supplementary material.

## Ethics statement

Synovial tissue from 3 patients of meniscus injury and 3 of OA were collected from Affiliated Hospital of the Guangxi University of Chinese Medicine. All patients critically read and signed the informed consent form (Ethics 2022-030-02), which was approved by the ethics committee of Affiliated Hospital of the Guangxi University of Chinese Medicine. The research followed the guidelines of the 1975 Declaration of Helsinki.

## Author contributions

BX and JN designed this study. BX drafted the manuscript. JN, GL and PH performed the statistical analysis and contributed equally to the work. YH and LC revised this manuscript. All authors contributed to the article and approved the submitted version.

## References

[1] Prieto-Potin I Largo R ROman-Blas JA Herrero-Beaumont G Walsh DA. Characterization of multinucleated giant cells in synovium and subchondral bone in knee osteoarthritis and rheumatoid arthritis. BMC Musculoskelet Disord (2015) 16:226. doi: 10.1186/s12891-015-0664-5 26311062 PMC4550054

[2] Han D Fang Y Tan X Jiang H Gong X Wang X. The emerging role of fibroblast-like synoviocytes-mediated synovitis in osteoarthritis: An update. J Cell Mol Med (2020) 24(17):9518–32. doi: 10.1111/jcmm.15669 PMC7520283 32686306

[3] Michelucci A Cordes T Ghelfi J Pailot A Reiling N Goldmann O. Immune-responsive gene 1 protein links metabolism to immunity by catalyzing itaconic acid production. Proc Natl Acad Sci U.S.A. (2013) 110(19):7820–5. doi: 10.1073/pnas.1218599110 PMC3651434 23610393

[4] Katz JN Arant KR Loeser RF. Diagnosis and treatment of hip and knee osteoarthritis: A review. JAMA (2021) 325(6):568–78. doi: 10.1001/jama.2020.22171 PMC8225295 33560326

[5] Faust HJ Zhang H Han J Wolf MT Jeon OH Sadtler K. IL-17 and immunologically induced senescence regulate response to injury in osteoarthritis. J Clin Invest (2020) 130(10):5493–507. doi: 10.1172/JCI134091 PMC7524483 32955487

[6] Ma Y Yang H Zong X Wu J Ji X Liu W. Artificial M2 macrophages for disease-modifying osteoarthritis therapeutics. Biomaterials (2021) 274:120865. doi: 10.1016/j.biomaterials.2021.120865 33991950

[7] Chen Z Ma Y Li X Deng Z Zheng M Zheng Q. The immune cell landscape in different anatomical structures of knee in osteoarthritis: A gene expression-based study. BioMed Res Int (2020) 2020:1-21. doi: 10.1155/2020/9647072 PMC7106908 32258161

[8] Rajandran SN Ma CA Tan JR Liu J Wong SBS Leung YY. Exploring the association of innate immunity biomarkers with MRI features in both early and late stages osteoarthritis. Front Med (Lausanne) (2020) 7:554669. doi: 10.3389/fmed.2020.554669 33282885 PMC7689194

[9] Tsvetkov P Coy S Petrova B Dreishpoon M Verma A Abdusamad M. Copper induces cell death by targeting lipoylated TCA cycle proteins. Science (2022) 375:1254–61:6586. doi: 10.1126/science.abf0529 PMC9273333 35298263

[10] Tan HY Wang N Zhang C Chan YT Yuen MF Feng Y. Lysyl oxidase-like 4 fosters an immunosuppressive microenvironment during hepatocarcinogenesis. Hepatology (2021) 73(6):2326–41. doi: 10.1002/hep.31600 PMC8251926 33068461

[11] Choi BY Jang BG Kim JH Seo JN Wu G Sohn M. Copper/zinc chelation by clioquinol reduces spinal cord white matter damage and behavioral deficits in a murine MOG-induced multiple sclerosis model. Neurobiol Dis (2013) 54:382–91. doi: 10.1016/j.nbd.2013.01.012 23360710

[12] Cheng Q Chen X Wu H Du Y. Three hematologic/immune system-specific expressed genes are considered as the potential biomarkers for the diagnosis of early rheumatoid arthritis through bioinformatics analysis. J Transl Med (2021) 19(1):18. doi: 10.1186/s12967-020-02689-y 33407587 PMC7789535

[13] Kaminska JA. A random forest partition model for predicting NO2 concentrations from traffic flow and meteorological conditions. Sci Total Environ (2019) 651(Pt 1):475–83. doi: 10.1016/j.scitotenv.2018.09.196 30243167

[14] Huang S Cai N Pacheco PP Narrandes S Wang Y Xu W. Applications of support vector machine (SVM) learning in cancer genomics. Cancer Genomics Proteomics (2018) 15(1):41–51. doi: 10.21873/cgp.20063 29275361 PMC5822181

[15] Wang L Wei S Zhou B Wu S. A nomogram model to predict the venous thromboembolism risk after surgery in patients with gynecological tumors. Thromb Res (2021) 202:52–8. doi: 10.1016/j.thromres.2021.02.035 33735691

[16] Zhang Y Liu N Wang S. A differential privacy protecting K-means clustering algorithm based on contour coefficients. PloS One (2018) 13 11:e0206832. doi: 10.1371/journal.pone.0206832 30462662 PMC6248925

[17] Ahn SH Yang HY Tran GB Kwon J Son KY Kim S. Interaction of peroxiredoxin V with dihydrolipoamide branched chain transacylase E2 (DBT) in mouse kidney under hypoxia. Proteome Sci (2015) 13:4. doi: 10.1186/s12953-014-0061-2 25670924 PMC4323032

[18] Abdul SZ Cero C Pierce AE Lea HJ Zhu KY Liu N. Combining a beta3 adrenergic receptor agonist with alpha-lipoic acid reduces inflammation in male mice with diet-induced obesity. Obes (Silver Spring) (2022) 30(1):153–64. doi: 10.1002/oby.23309 PMC8692380 34825496

[19] Mayr JA Feichtinger RG Tort F Ribes A Sperl W. Lipoic acid biosynthesis defects. J Inherit Metab Dis (2014) 37(4):553–63. doi: 10.1007/s10545-014-9705-8 24777537

[20] Frommer KW Hasseli R Schaffler A Lange U Rehart S Steinmeyer J. Free fatty acids in bone pathophysiology of rheumatic diseases. Front Immunol (2019) 10:2757. doi: 10.3389/fimmu.2019.02757 31849953 PMC6901602

[21] Rowland EA Snowden CK Cristea IM. Protein lipoylation: an evolutionarily conserved metabolic regulator of health and disease. Curr Opin Chem Biol (2018) 42:76–85. doi: 10.1016/j.cbpa.2017.11.003 29169048 PMC5965299

[22] Tang Q Guo Y Meng L Chen X. Chemical tagging of protein lipoylation. Angew Chem Int Ed Engl (2021) 60(8):4028–33. doi: 10.1002/anie.202010981 33174356

[23] Nie X Chen H Xiong Y Chen J Liu T. Anisomycin has a potential toxicity of promoting cuproptosis in human ovarian cancer stem cells by attenuating YY1/lipoic acid pathway activation. J Cancer (2022) 13(14):3503–14. doi: 10.7150/jca.77445 PMC9723990 36484005

[24] Smolle M Lindsay JG. Molecular architecture of the pyruvate dehydrogenase complex: bridging the gap. Biochem Soc Trans (2006) 34(Pt 5):815–8:no. doi: 10.1042/BST0340815 17052205

[25] Saunier E Benelli C Bortoli S. The pyruvate dehydrogenase complex in cancer: An old metabolic gatekeeper regulated by new pathways and pharmacological agents. Int J Cancer (2016) 138(4):809–17. doi: 10.1002/ijc.29564 25868605

[26] Langston PK Nambu A Jung J Shibata M Aksoylar HI Lei J. Glycerol phosphate shuttle enzyme GPD2 regulates macrophage inflammatory responses. Nat Immunol (2019) 20(9):1186–95. doi: 10.1038/s41590-019-0453-7 PMC6707851 31384058

[27] Park S Baek IJ Ryu JH Chun CH Jin EJ. PPARalpha-ACOT12 axis is responsible for maintaining cartilage homeostasis through modulating de novo lipogenesis. Nat Commun (2022) 13(1):3. doi: 10.1038/s41467-021-27738-y 34987154 PMC8733009

[28] Echeverri RNP Mohan V Wu J Scott S Kreamer M Benej M. Dynamic regulation of mitochondrial pyruvate metabolism is necessary for orthotopic pancreatic tumor growth. Cancer Metab (2021) 9(1):39. doi: 10.1186/s40170-021-00275-4 34749809 PMC8577026

[29] Feng X Zhang L Xu S Shen AZ. ATP-citrate lyase (ACLY) in lipid metabolism and atherosclerosis: An updated review. Prog Lipid Res (2020) 77:101006. doi: 10.1016/j.plipres.2019.101006 31499095

[30] Patel MS Nemeria NS Furey W Jordan F. The pyruvate dehydrogenase complexes: structure-based function and regulation. J Biol Chem (2014) 289(24):16615–23. doi: 10.1074/jbc.R114.563148 PMC4059105 24798336

[31] Stifel U Wolfschmitt EM Vogt J Wachter U Vettorazzi S Tews D. Glucocorticoids coordinate macrophage metabolism through the regulation of the tricarboxylic acid cycle. Mol Metab (2022) 57:101424. doi: 10.1016/j.molmet.2021.101424 34954109 PMC8783148

[32] Cordes T Wallace M Michelucci A Divakaruni AS Sapcariu SC Sousa C. Immunoresponsive gene 1 and itaconate inhibit succinate dehydrogenase to modulate intracellular succinate levels. J Biol Chem (2016) 291(27):14274–84. doi: 10.1074/jbc.M115.685792 PMC4933182 27189937

[33] Lampropoulou V Sergushichev A Bambouskova M Nair S Vincent EE Loginicheva E. Itaconate links inhibition of succinate dehydrogenase with macrophage metabolic remodeling and regulation of inflammation. Cell Metab (2016) 24(1):158–66. doi: 10.1016/j.cmet.2016.06.004 PMC5108454 27374498

[34] Higgins M Hams E Szpyt J Runtsch MC King MS McGouran JF. Itaconate is an anti-inflammatory metabolite that activates Nrf2 via alkylation of KEAP1. Nature (2018) 556(7699):113–7. doi: 10.1038/nature25986 PMC6047741 29590092

[35] Strushkevich N MacKenzie F Cherkesova T Grabovec I Usanov S Park HW. Structural basis for pregnenolone biosynthesis by the mitochondrial monooxygenase system. Proc Natl Acad Sci U.S.A. (2011) 108(25):10139–43. doi: 10.1073/pnas.1019441108 PMC3121847 21636783

[36] Zhang Z Ma Y Guo X Du Y Zhu Q Wang X. FDX1 can impact the prognosis and mediate the metabolism of lung adenocarcinoma. Front Pharmacol (2021) 12:749134. doi: 10.3389/fphar.2021.749134 34690780 PMC8531531

[37] Colliou N Ge Y Sahay B Gong M Zadeh M Owen JL. Commensal Propionibacterium strain UF1 mitigates intestinal inflammation via Th17 cell regulation. J Clin Invest (2017) 127(11):3970–86. doi: 10.1172/JCI95376 PMC5663347 28945202

[38] Belluzzi E Stocco E Pozzuoli A Granzotto M Porzionato A Vettor R. Contribution of infrapatellar fat pad and synovial membrane to knee osteoarthritis pain. BioMed Res Int (2019) 2019:6390182. doi: 10.1155/2019/6390182 31049352 PMC6462341

[39] You L Chen L Pan L Peng Y Chen J. SOST gene inhibits osteogenesis from adipose-derived mesenchymal stem cells by inducing th17 cell differentiation. Cell Physiol Biochem (2018) 48(3):1030–40. doi: 10.1159/000491971 30041240

[40] Sakkas LI Scanzello C Johanson N Burkholder J Mitra A Salgame P. T cells and T-cell cytokine transcripts in the synovial membrane in patients with osteoarthritis. Clin Diagn Lab Immunol (1998) 5(4):430–7. doi: 10.1128/CDLI.5.4.430-437.1998 PMC95595 9665944

[41] Sarkar S Justa S Brucks M Endres J Fox DA Zhou X. Interleukin (IL)-17A, F and AF in inflammation: a study in collagen-induced arthritis and rheumatoid arthritis. Clin Exp Immunol (2014) 177 3:652–61. doi: 10.1111/cei.12376 PMC4137849 24813051

[42] Li YS Luo W Zhu SA Lei GH. T cells in osteoarthritis: alterations and beyond. Front Immunol (2017) 8:356. doi: 10.3389/fimmu.2017.00356 28424692 PMC5371609

[43] Liu H Luo T Tan J Li M Guo J. Osteoimmunology offers new perspectives for the treatment of pathological bone loss. Curr Pharm Des (2017) 23(41):6272–8. doi: 10.2174/1381612823666170511124459 28494718

[44] Kim HR Kim KW Kim BM Won JY Min HK Lee KA. Regulation of th17 cytokine-induced osteoclastogenesis via SKI306X in rheumatoid arthritis. J Clin Med (2019) 8(7):1012. doi: 10.3390/jcm8071012 31295961 PMC6678573

[45] Iwakura Y Nakae S Saijo S Ishigame H. The roles of IL-17A in inflammatory immune responses and host defense against pathogens. Immunol Rev (2008) 226:57–79. doi: 10.1111/j.1600-065X.2008.00699.x 19161416

[46] Askari A Naghizadeh MM Homayounfar R Shahi A Afsarian MH Paknahad A. Increased serum levels of IL-17A and IL-23 are associated with decreased vitamin D3 and increased pain in osteoarthritis. PloS One (2016) 11(11):e0164757. doi: 10.1371/journal.pone.0164757 27820818 PMC5098728

[47] Shan Y Qi C Liu Y Gao H Zhao D Jiang Y. Increased frequency of peripheral blood follicular helper T cells and elevated serum IL21 levels in patients with knee osteoarthritis. Mol Med Rep (2017) 15(3):1095–102. doi: 10.3892/mmr.2017.6132 PMC5367351 28112376

[48] Monasterio G Castillo F Rojas L Cafferata EA Alvarez C Carvajal P. Th1/Th17/Th22 immune response and their association with joint pain, imagenological bone loss, RANKL expression and osteoclast activity in temporOmandibular joint osteoarthritis: A preliminary report. J Oral Rehabil (2018) 45(8):589–97. doi: 10.1111/joor.12649 29761933

[49] Miyazaki Y Nakayamada S Kubo S Nakano K Iwata S Miyagawa I. Th22 cells promote osteoclast differentiation via production of IL-22 in rheumatoid arthritis. Front Immunol (2018) 9:2901. doi: 10.3389/fimmu.2018.02901 30619268 PMC6295478

[50] Killock D. Osteoimmunology: Could inhibition of IL-1 and TNF improve healing of meniscal lesions and prevent the development of osteoarthritis? Nat Rev Rheumatol (2011) 8(1):4. doi: 10.1038/nrrheum.2011.191 22193854

[51] Stannus O Jones G Cicuttini F Parameswaran V Quinn S Burgess J. Circulating levels of IL-6 and TNF-alpha are associated with knee radiographic osteoarthritis and knee cartilage loss in older adults. Osteoarthritis Cartilage (2010) 18(11):1441–7. doi: 10.1016/j.joca.2010.08.016 20816981

[52] Cai L Yin JP Starovasnik MA Hogue DA Hillan KJ Mort JS. Pathways by which interleukin 17 induces articular cartilage breakdown in vitro and in *vivo* . Cytokine (2001) 16(1):10–21. doi: 10.1006/cyto.2001.0939 11669582

[53] Haseeb A Haqqi TM. Immunopathogenesis of osteoarthritis. Clin Immunol (2013) 146(3):185–96. doi: 10.1016/j.clim.2012.12.011 PMC4015466 23360836

[54] Jin M Shi C Li T Wu Y Hu C Huang G. Solasonine promotes ferroptosis of hepatoma carcinoma cells via glutathione peroxidase 4-induced destruction of the glutathione redox system. BioMed Pharmacother (2020) 129:110282. doi: 10.1016/j.biopha.2020.110282 32531676

[55] Afonso V Champy R Mitrovic D Collin P Lomri A. Reactive oxygen species and superoxide dismutases: role in joint diseases. Joint Bone Spine (2007) 74(4):324–9. doi: 10.1016/j.jbspin.2007.02.002 17590367

[56] Mathy-Hartert M Hogge L Sanchez C Deby-Dupont G Crielaard JM Henrotin Y. Interleukin-1beta and interleukin-6 disturb the antioxidant enzyme system in bovine chondrocytes: a possible explanation for oxidative stress generation. Osteoarthritis Cartilage (2008) 16(7):756–63. doi: 10.1016/j.joca.2007.10.009 18291685

[57] Tilg H Dinarello CA Mier JW. IL-6 and APPs: anti-inflammatory and immunosuppressive mediators. Immunol Today (1997) 18(9):428–32. doi: 10.1016/s0167-5699(97)01103-1 9293158

